# Long Non-Coding RNAs (lncRNAs) in Response and Resistance to Cancer Immunosurveillance and Immunotherapy

**DOI:** 10.3390/cells10123313

**Published:** 2021-11-26

**Authors:** Giasemi C. Eptaminitaki, Nora Wolff, Dimitris Stellas, Konstantinos Sifakis, Stavroula Baritaki

**Affiliations:** 1 Laboratory of Experimental Oncology, Division of Surgery, School of Medicine, University of Crete, GR-71003 Heraklion, Greece; eptaminitaki.giasemi@gmail.com (G.C.E.); nora.wolff.klbr@gmail.com (N.W.); kostasifakis@outlook.com (K.S.); 2 Institute of Chemical Biology, National Hellenic Research Foundation (NHRF), 48 Vassileos Constantinou Ave., GR-11635 Athens, Greece; dstellas@eie.gr

**Keywords:** long non-coding RNAs, cancer, tumor resistance, immunotherapy, immunosurveillance

## Abstract

Long non-coding RNAs (lncRNAs) are critical regulatory elements in cellular functions in states of both normalcy and disease, including cancer. LncRNAs can influence not only tumorigenesis but also cancer features such as metastasis, angiogenesis and resistance to chemo-and immune-mediated apoptotic signals. Several lncRNAs have been demonstrated to control directly or indirectly the number, type and activities of distinct immune cell populations of adaptive and innate immunities within and without the tumor microenvironment. The disruption of lncRNA expression in both cancer and immune cells may reflect alterations in tumor responses to cancer immunosurveillance and immunotherapy, thus providing new insights into lncRNA biomarker-based prognostic and therapeutic cancer assessment. Here we present an overview on lncRNAs’ functions and underlying molecular mechanisms related to cancer immunity and conventional immunotherapy, with the expectation that any elucidations may lead to a better understanding and management of cancer immune escape and response to current and future immunotherapeutics.

## 1. Introduction

Cancer remains the second leading cause of mortality worldwide after cardiovascular diseases [1]. Cancer as a genetic disorder has its molecular base in the accumulation of multiple mutations, mostly in genes that control directly or indirectly cell survival and proliferation, as well as the inability of the host immune-surveillance mechanisms to recognize and kill the cancer cells [2,3,4]. Although novel chemo- and immuno-therapeutics have prolonged overall patient survival and quality of life, disease relapses frequently occur due to failure of long lasting anti-tumor responses [5,6,7]. Most tumor types, as they progress, may further acquire additional genetic alterations that confer to the formation of cancer phenotypes resistant to conventional treatment modalities and highly potent for immune-evasion [3]. The list of molecular mechanisms that have so far been identified as responsible for the acquisition of tumor resistance to endogenous immune-mediated cytotoxicity and conventional chemo-and immune-therapeutics is quite long, and it is constantly being enriched [8,9,10,11]. Given the complexity of these mechanisms and their tumor specificity that is often observed, the molecular basis of resistance and the ways to overcome it need to be better elucidated and explored on multiple levels. As such, there is enormous space for research on identifying novel molecular signatures of tumor unresponsiveness to external and internal apoptotic signals that may be putative candidates for therapeutic targeting.

A large part of the human genome is transcribed into long non-coding RNAs (lncRNAs), which are defined as >200 nt long transcripts [12]. While more than 90% of lncRNAs lack protein-coding potential, it has been reported that some transcripts might encode short open reading frames (ORFs, length <300 nt), which could be translated into small peptides [13]. Only a few lncRNAs have been detected to act bi-functionally, both as RNA and peptides [13]. Although for many years the function of lncRNAs remained largely unknown, with many considering them as ‘genetic debri’, we now know that these RNA molecules can play a catalytic role in regulating protein-coding gene expression at epigenetic, transcriptional, post-transcriptional, translational and post-translational levels [12,14]. LncRNAs may interact with DNA regions and other cellular RNA species, such as mRNAs and miRNAs, thus affecting critical sites and accessibility to factors necessary for their function [15,16]. 

Decoy lncRNAs for example are known to interact directly with transcription factors [12,14,15,16], while others have been found to be involved in epigenetic modifications of the genome, genetic imprinting (e.g., lncRNAs *Xist* and *H19*) [12], changes in the conformation of chromatin and mRNA splicing, degradation and translation [12,14,16]. LncRNAs may further interact with various proteins, regulating indirectly gene expression and directly intracellular signal transduction pathways [15]. Guide lncRNAs, such as *Air*, *CCND1* and *linc-p21*, have the ability to attach to enzymes or regulatory proteins, such as chromatin-modifying complexes, leading them to their substrate or DNA targets, respectively [15,16,17]. In addition, binding of several proteins to scaffold lncRNAs can facilitate their interaction with other proteins, or allow the formation of ribonucleoprotein complexes, which in turn can activate or inhibit gene expression [14,15]. One such example is the telomerase RNA (*TERC*) that acts as a scaffold to telomerase reverse transcriptase (TERT) for the synthesis of telomeric DNA repeats [17]. On the other hand, signaling lncRNAs are expressed in response to certain conditions, such as cellular damage, and may regulate signaling pathways, gene expression and other cell functions [15,16]. Characteristically, we can mention the lncRNAs *linc-p21* and *PANDA* known to be induced by DNA damage, the *COLDAIR* and *COOLAIR* induced by cold, and *ROR*, a reprogramming-induced lncRNA that has been directly associated with cell pluripotency [17].

Alterations in lncRNA expression levels have been lately associated with many disorders including cardiovascular and neurodegenerative diseases [18,19]. Many lncRNAs have been reported to be involved in the regulation of the cell cycle, especially by targeting cyclins, cyclin-dependent kinases (CDKs) and/or CDK inhibitors [16]. Mutations, single nucleotide polymorphisms (SNPs), copy-number alterations or epigenetic modifications within the non-coding genome, increase the risk of altering lncRNA expression levels, and thus lead to cell cycle deregulation and potentially to carcinogenesis [19,20,21]. As such, the modified expression of some lncRNAs have been identified as hallmarks of many cancer properties, including uncontrolled proliferation, viability, immortality, genomic instability, motility, invasion, metastasis, angiogenesis and drug resistance in a cell type-specific manner [21]. 

In the following paragraphs we overview the literature on lncRNA molecules reported to interfere with tumor resistance to endogenous immune-mediated cytotoxicity and immunotherapy, with special focus on the wide range of the underlying molecular mechanisms of their action on cancer resistance regulation.

## 2. Mechanisms of Tumor Immune Evasion and Resistance to Immunotherapy

The anti-tumor responses are mediated by activation of both innate and adaptive immunities, whose efficacies largely depend on the tumor status (initiated vs progressed disease), the tumor composition in neoantigens and the potential of the involved immune cells to be properly activated and highly aggressive against cancer cells [22]. The main components of the adaptive immune responses, the CD8+ and CD4+ T cell populations, are both armed with T cell receptors (TCRs), which can recognize respectively major histocompatibility complex (MHC) classI/ or MHC classII/tumor-derived antigen complexes (“stimulating complex”) in a highly specific and selective manner. Naïve CD8+ and CD4+ T cells are activated by mature dendritic cells (DCs), the professional tumor antigen presenting cells (APCs), to become effector cells through TCR binding to “stimulating complexes” on DCs (priming phase). Effector CD8+ T cells in turn are targeted to recognize and interact with cancer cells curing on their surface the same ‘’stimulating complex’’ and forcing them to undergo apoptosis (effector phase). Effector CD4+ T cells can either synergize with DCs in activating naive cytotoxic CD8+ T cells or interact with B cells inducing them to differentiate into immunoglobulin producing plasma cells. Under certain circumstances, as in the case of increased accumulation of transforming growth factor beta (TGF-β) secreted by tumor cells into the tumor microenvironment (TME), infiltrated effector CD4+ T cells can be “polarized” into T regulatory cells (Tregs), which perform an immunosuppressive function. Although, natural killer (NK) cells derive from lymphocyte progenitor cells as well, they belong to the innate immune system, since their cytotoxic activity is generally directed against opsonized target cells, in a non-specific manner [7,23,24].

**Figure 1:**
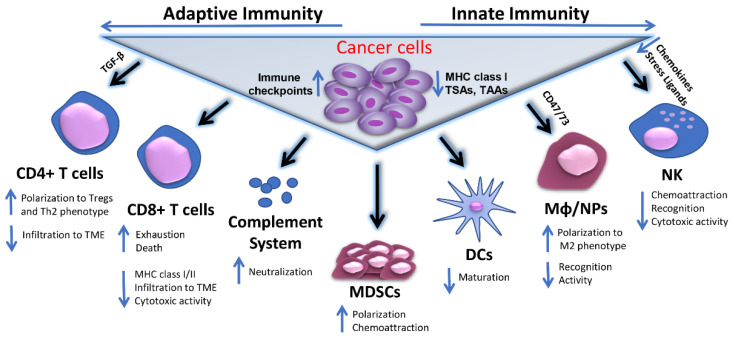
Mechanisms of tumor immune evasion. Cancer cells escape host immunosurveillance by multiple mechanisms associated either with their phenotypic characteristics or with different immune cell populations of adaptive and innate immunity involved in the induction or suppression of a successful anti-tumor immune response. These mechanisms may include reduced expression of MHC class I, TAA, TSAs in cancer cells and/or upregulation of inhibitory immune checkpoint molecules on their surface. Alternatively, tumor cells may affect CD4+ and CD8+ T cell infiltration to TME by secreting factors, like TGF-β, while they can trigger the polarization of effector CD4+ T cells to Treg and/or Th2 phenotypes. In parallel, they can promote CTL exhaustion and apoptosis, accompanied by reduced CTL-associated expression of MHC class I/II complexes and cytotoxic activities. Concomitantly, the functions of innate immune cell populations, including Mφ, NPs and NK cells are significantly diminished, either due to accumulation of suppressing molecules like CD47 and CD73, or lack of potent chemoattractors and stress-related ligands, or inefficient target recognition. Cancer cells may also negatively interfere with DC maturation and complement dependent cytotoxicity (CDC), mainly by expression of neutralizing complement regulatory proteins. The immunosuppressive effects in TME are further augmented by cancer cell-mediated MDSCs recruitment and polarization to immunosuppressive cell phenotypes. Abbreviations used: MHC I/II, Major histocompatibility complex classI/or classII; TAA, Tumor associated antigens; TSA, Tumor specific antigens; MDSCs, Myeloid-derived suppressor cells; DCs, Dendritic cells; Mφ, Macrophages; NPs, Neutrophils; NK, Natural killers; TME, Tumor microenvironment; Tregs, T regulatory cells; Th2, Type 2 helper; CTL, Cytotoxic T lymphocyte; TGF-β, transforming growth factor beta.

Cancer cells evolve multiple mechanisms to elude detection and destruction by the innate and adaptive immune system [25]. Multiple modifications on cancer cell membranes such as underexpression of MHC class I antigens, upregulation of inhibitory immune checkpoint molecules, like programmed death-ligand 1 (PD-L1), and reduced presentation of tumor associated and tumor specific antigens (TAA and TSA) are among the most prevalent mechanisms associated with tumor escape from host adaptive immune-surveillance [25,26]. Malignant cells may further evade recognition by macrophages, neutrophils and other innate immune cells via induction of “don’t eat me” signals (e.g., CD47, CD73), while by downregulating chemokines and stress ligands tumor cells may poorly respond to attraction of and recognition by NK cells. In addition, components of the humoral anti-tumor response such as the complement system-mediated cytotoxicity is counteracted by expression of neutralizing complement regulatory proteins (e.g., CD46/55/59) [27]. The above mechanisms of resistance are summarized schematically in Figure 1.

According to the theory of cancer immunoediting, the acquisition of the immuno-resistant cancer cell phenotype is the result of adaptation to the unstable immune environment that exerts evolutionary pressure on the mutated cells, along with the high tumor heterogeneity and genomic instability that make a small fraction of cancer cells able to bypass host immune surveillance. So while at first glance the relationship between the immune system and cancer cells appears one sided, with the immune system the force for tumor selection, new evidence emerges that this relationship may be reciprocal with the cancer cell being able to achieve possible epigenetic changes in the genome of immune cells during oncogenesis and tumor progression that weaken the anti-tumor response, thus leading to tumor immune evasion [28]. 

Given that the efficacies of both passive and active immunotherapeutic approaches mostly depend on setting in motion a general or specific immune response by activating either immune system components, like complement proteins, and/or a wide range of immune cells, it is clear that immunotherapy failure might be at least partially attributed to its inability to activate a successful anti-tumor immune response that consequently may lead or sustain cancer immune evasion. To this end, cancer resistance to both host immune surveillance and immunotherapy may share common underlying mechanisms [25]. It is quite common, however, for the tumor resistance to immunotherapy to be mediated by acquired epigenetic changes in immunotherapeutic targets or by their complete elimination, thus allowing tumors to bypass the therapeutic effect, while activating alternative signaling cascades to sustain the target function. These alterations often occur in antibody targets, like growth receptors, that may develop mutations to escape antibody recognition and/or even mobilize other members of the same family to maintain the intracellular transduction of the growth signal [25].

The involvement of lncRNAs in the bidirectional communication between immune system and cancer cells is less understood [29,30]. LncRNAs have a quite close relationship with the early regulation stages of immune responses, while their expression seems to be highly ‘personalized’ to different tumors, therefore affecting the individual anti-tumor responses, as will be discussed in the following paragraphs. 

## 3. LncRNAs and Cell-Mediated Anti-Tumor Immunity

The expression patterns of lncRNAs are precise, highly cell type-specific and context-specific, as well as dependent on the stage of development and cell differentiation [31]. This is important for the smooth function of the immune system, which relies on the self-renewal and differentiation of hematopoietic stem cells (HSCs), where lncRNAs might serve as mediators between extracellular signals and the activity of transcriptional factors that determine the cell differentiation path and fate [32]. Common lymphocyte progenitor cells are differentiated into specific NK, B and T cell lineages, each expressing a different combination of lncRNAs. LncRNAs have been reported to play a significant role during the development, differentiation and activation of CD8+ T cells [33,34,35,36], CD4+ T cells [33,34,35,36,37] and NK cells [33,35,36]. 

Dysregulated patterns of lncRNA expression in tumor cells and different immune cell populations, including populations of both innate and adaptive immunities, have been directly and indirectly associated with the survival, activation and cytotoxic potency of effector immune cells involved in the anti-tumor responses. In the following paragraphs we discuss the pleiotropic effects of different lncRNAs in tumor response to innate and adaptive immunity, emphasizing on the nature of lncRNA transcriptome in the involved immune cell populations.

### 3.1. Regulation of Anti-Tumor CTL Response by lncRNAs

#### 3.1.1. Immune Cell-Associated lncRNAs

LncRNAs expressed in both immune and cancer cells have been reported to modulate the anti-tumor cytotoxic T lymphocyte (CTL) responses via various molecular mechanisms. Table 1 contains a list of such lncRNAs.

- CD4+ T cell-expressed lncRNAs

The lncRNAs small nucleolar RNA host gene 1 (*lnc-SNHG1*), epidermal growth factor receptor (*lnc-EGFR*) and insulin receptor precursor (*lnc-INSR*) have been reported to reduce the cytotoxic activities of CD8+ T cells within the TME, by accelerating the polarization of tumor infiltrated CD4+ T lymphocytes (TILs) into regulatory Tregs (CD4+/CD25+/Foxp3+ T cells) [38,39,40]. Specifically, in breast cancer (BC) models, upregulation of *lnc-SNHG1* in CD4+ TILs promotes their differentiation into Tregs by interacting with miR-448, a negative regulator of indoleamine 2,3-dioxygenase (IDO). Induction of IDO by *lnc-SNHGI*-mediated inhibition of miR-448 results in increased CD4+ T cell differentiation into Tregs, and eventually in suppression of CTLs and induction of their apoptotic death [38]. Concomitantly, immune evasion of hepatocellular carcinoma (HCC) is supported by the *lnc-EGFR*, whose upregulation in tumor infiltrating CD4+ T cells also leads to their polarization into Treg cells. *Lnc-EGFR* binds to EGFR, thus sustaining its preservation and activation. EGFR activation in turn induces the AP1/NF-AT1 axis, which acts as a forward-feedback loop reinforcing *lnc-EGFR* expression and enhancing the transcription of EGFR and forkhead box protein P3 (FOXP3) genes. While their number increases, Tregs downregulate the action and propagation of effector T-cells within the TME, resulting in immunosuppression, toleration and progression of HCC cells [39]. On the same motif, abnormal overexpression of *lnc-INSR* in bone marrow infiltrated CD4+ T cells in pediatric T cell acute lymphoblastic leukemia (T-ALL) modulates the immune microenvironment and results in Treg increase and a decline in CTLs, thus allowing the tolerance of malignant cells in the bone marrow. Being co-localized with the INSR both in the membrane and the cytoplasm, *lnc-INSR* restrains the ubiquitination of INSR, thus sustaining INSR activation and stability. This leads to an enhanced activation of the PI3K/AKT-signaling pathway that promotes Treg cell differentiation and an immunosuppressive microenvironment [40]. 

Accordingly, elevated levels of long intergenic noncoding RNA POU3F3 (*linc-POU3F3*) in gastric cancer (GC) patient-derived Tregs augments Treg distribution, through modulation of TGF-β signaling pathway [41], while lncRNA *Flatr* enhances the immunosuppressive function of Tregs by boosting FOXP3 expression [42]. In contrast, lncRNA Foxp3 long intergenic noncoding RNA (*Flicr*) negatively regulates FOXP3 gene loci accessibility and expression, resulting in impaired Treg activity [43]. Moreover, in CD4+ T cells the long intergenic noncoding RNA musculoaponeurotic fibrosarcoma-4 (*linc-MAF-4*)-mediated regulation of musculoaponeurotic fibrosarcoma (MAF) transcriptional activity, through recruitment of chromatin modifiers, promotes the differentiation of T cells towards a Th2 phenotype, which is known to mainly trigger the humoral instead of the cellular arm of the adaptive immunity [44]. Similarly, induced expression of lncRNA serum/glucocorticoid regulated kinase 1 (*lnc-SGK1*) in gastric cancer-derived T cells by Helicobacter pylori infection and a high salt diet forces type 2 helper (Th2) and type 17 helper (Th17) phenotypes, while reduces type 1 helper (Th1) differentiation, via SGK1/Jun-B signaling pathway [45].

- CD8+ T cell-expressed lncRNAs

In lung and breast cancer models overexpression of the NF-κB-interacting lncRNA (*NKILA*) in tumor-infiltrating CTLs and Th1 cells leads to their activation-induced cell death (AICD) through attenuation of NF-κB-dependent anti-apoptotic gene transcription. Briefly, *NKILA* transcriptional activity is significantly induced during the late-sensitive phase of T cell activation, after TCR stimulation triggers the recruitment of histone acetyltransferases to the *NKILA* promoter, which allow chromatin opening and binding of the transcriptional activator of *NKILA*, signal transducer and activator of transcription 1 (STAT1). While becoming upregulated, *NKILA* binds to NF-κB and represses its activity on downstream anti-apoptotic target genes, thus forcing effector CD8+ T cells to undergo AICD and tumor cells to escape CTL-mediated cytotoxicity [46]. In addition, a recent study exploiting the fundamental mechanisms by which CD8+ T cells are regulated in response to pathogens and potentially cancer, demonstrated evidence for a critical role of lncRNA *Morrbid* in CD8+ T cell expansion, survival and effector function by modulating the expression of the pro-apoptotic factor Bcl-2-like protein 11 (Bcl2111) and the activity of the PI3K-AKT signaling pathway [47]. Concomitantly, in colorectal cancer (CRC) xenograft mouse models induction of lncRNA *GM16343* in IL-36-stimulated mouse CD8+ T cells increased their interferon gamma (IFN-γ) secreting ability in the TME and reduced tumor diameter, thus suggesting a critical role of lncRNA *GM16343* in reinforcing the CD8+ T cell-mediated anti-tumor immune response [48].

- DC-expressed lncRNAs

DCs, as professional APCs, play a pivotal role in regulating the balance between CD8+ T cell-mediated immunity and tolerance to tumor antigens. DCs activate CD8+ T cells by cross-presenting non-self-antigens, a process known as cross priming. Cross priming is thought to be critical in generating potent anti-tumor CTL responses [49]. LncRNA dysregulation in DCs can modulate their anti-tumor function by affecting their tumor infiltration, differentiation and metabolism, as well as their ability to cross-prime CD8+ T cell-mediated responses [50]. A characteristic example of a DC-associated lncRNA is the *lnc-DC*, which promotes DCs’ differentiation and increases their ability to stimulate T cell activation by activating STAT3 [51]. Studies on *lnc-DC* silencing, upon hepatitis B virus (HBV) infection, showed that *lnc-DC* inhibits DCs’ maturation and controls virally induced immune responses. The underlying mechanism of action includes *lnc-DC*-mediated reduction in IFN-γ, IL-6, IL-12 and tumor necrosis factor alpha (TNF-α) secretion and increase in IL-1β concentration in DCs, via targeting the TLR9/STAT3 signaling pathway [52]. *Lnc-Dpf3* induction in DCs inhibits DC migration and glycolytic metabolism, by decreasing the hypoxia inducible factor 1 subunit alpha (HIF-1a)-mediated glycolysis [53].

- Macrophage-expressed lncRNAs

Macrophages act both as anti-tumorigenic, when in M1 stage and pro-tumorigenic, when in M2 stage [54]. M1 macrophages secrete pro-inflammatory cytokines and chemokines, such as IL-6, IL-12 and TNF-α, as well as act as APCs. In contrast, M2 macrophages secrete arginase 1 (Arg1), IL-10 and TGF-β suppressing CTL immune responses and promoting immune suppression [55]. Many studies have shown that lncRNAs affect tumor-associated macrophages (TAMs) by regulating M2 macrophage polarization. LncRNAs *GNAS-AS1*, *XIST*, *lnc-P21*, *ANCR* and *lnc-MM2P* are among the lncRNAs that induce M2 macrophage polarization in a plethora of cancers, via various mechanisms [56,57,58,59,60,61] (See Table 1). For example, expression of lncRNA GNAS antisense 1 (*GNAS-AS1*) in non-small cell lung cancer (NSCLC) and BC, enhances M2 macrophage polarization and consequently tumor immune-evasion through regulation of *GNAS-AS1*/MIR4319/NECAB3 and *GNAS-AS1*/miR-433-3p/GATA3 axes, respectively [56,57]. In addition, macrophage-derived exosomal lncRNA LIFR antisense 1 (*LIFR-AS1*) and Sbf2 antisense 1 (*Sbf2-AS1*) were also revealed to be critical for osteosarcoma (OS) and pancreatic cancer progression, by modulating the miR-29a/NFIA and miR-122-5p/XIAP signaling cascades, respectively [62,63]. In contrast, other lncRNAs, including NIFK antisense 1 (*NIFK-AS1*), cyclooxygenase-2 (*COX-2*) and colon cancer-associated transcript 1 (*CCAT1*), have been associated with negative regulation of M2 macrophage polarization [64,65,66,67]. LncRNA *NIFK-AS1*, expressed in endometrial cancer, mediates this negative action via targeting miR-146a [64], while HCC-expressed lncRNA *COX-2* by activating and inhibiting distinct classes of immune genes [65,66]. Finally, lncRNA *CCAT1* expressed in prostate cancer inhibits macrophage polarization to M2 stage and prevents cancer cell migration by targeting miR-148a/PKCζ signaling [67].

- MDSC-expressed LncRNAs

The expansion of myeloid-derived suppressor cells (MDSCs), a heterogenous group of immature immune cells from the myeloid lineage, has been associated with immunosuppressive effects in TME and cancer progression [68,69]. Most of the tumor-derived MDSCs have lost their ability to differentiate into mature granulocytes, monocytes and macrophages, while they accelerate tumor progression by dramatically blocking T cell-induced antitumor responses and enhancing Tregs through release of Arg1, reactive oxygen species (ROS) and inducible nitric oxide synthase (iNOs) [68,70,71,72]. Interestingly, recent studies in the peripheral blood of LC patients revealed that the percentage of MDSCs was negatively correlated with the percentage of Th1/CTL cells, thus suggesting that MDSCs might pose a suppressive effect not only to CTL responses, but also to CTL numbers [69,73]. Although, the involvement of lncRNAs in the regulation of the immunosuppressive function of MDSCs has recently begun to be exploited, early indications reveal a critical role for lncRNAs in MDSC expansion and function in cancer [71]. 

Upregulation of lncRNA C/EBPβ and C/EBP homologous protein (*CHOP*) in MDSCs induces expression of immunosuppressive factors, such as NO synthase 2, NADPH oxidase 2, Arg1 and cyclooxygenase-2, via interactions with *CHOP* and liver-enriched inhibitory protein (LIP), leading to activation of CCAAT-enhancer-binding protein (C/EBPB) and augmentation of tumor growth in many cancers [74]. Furthermore, lncRNAs pseudogene *Olfr29-ps1* and retinal noncoding RNA 3 (*RNCR3*) regulate MDSC differentiation and promote their immunosuppressive phenotype by modulating the *Olfr29-ps1*/miR-214-3p/MyD88/M6A regulatory network [75] and *RNCR3*/miR-185-5p/Chop pathway, respectively [76]. LncRNA pseudogene *Olfr29-ps1* also contributes to upregulation of both inflammatory and tumor-associated factors in the microenvironment of melanoma (MM) tumors [75]. Similarly, lncRNAs plasmacytoma variant translocation 1 (*Pvt1*) and RUNX1 overlapping RNA (*RUNXOR*) accelerated MDSC-mediated immunosuppression in LC in in vitro and in vivo models [77,78]. Conversely, other lncRNAs such as HOXA transcript antisense RNA myeloid-specific 1 (*HOTAIRM1*) and metastasis associated lung adenocarcinoma transcript 1 (*MALAT1*), have been reported to negatively affect the proportion of MDSCs in peripheral blood mononuclear cells (PBMCs) and downregulate their immunosuppressive activity in cancer [69,73]. High levels of *HOTAIRM1* in LC patient-derived MDSCs could inhibit the development of MDSCs and the expression of MDSC-associated suppressive molecules, via induction of homeobox A1 (HOXA1), a molecule known to delay tumor progression and enhance the anti-tumor immune response by downregulating the immunosuppressive activity of MDSCs [69]. Accordingly, in the same tumor model, lncRNA *MALAT1* was shown to play a direct role in inhibiting MDSC expansion, while accelerating CTL proportion within TME [73]. 

Overall, although for some of the aforementioned lncRNAs there is not yet direct association with a specific cancer type, their contribution in the regulation of immune cell properties and functions open new insights in exploring their role in the anti-tumor immunity.

#### 3.1.2. Cancer Cell-Associated lncRNAs

A plethora of lncRNAs overexpressed in tumor cells may also modify the survival and activities of effector immune cells, via similar mechanisms to those described above. A list of such RNAs is shown in Table 2.

Overexpression of lncRNA SRY-box transcription factor 5 (*lnc-sox5*) in CRC cells leads to upregulation of IDO1 enzyme, which stimulates the differentiation of Tregs and eventually their infiltration into the tumor microenvironment. Tregs along with *lnc-sox5* IDO1 upregulation may in turn suppress CD8+ T cell cytotoxicity and intensify their exhaustion by activation of Fas/FasL-mediated apoptosis [83]. Similarly, the long non-coding RNA for kinase activation (*LINK-A*), expressed in 25% of triple-negative breast cancer (ΤΝΒC) patients, is thought to be a negative regulator of APC and effector CD8+ T cell recruitment and infiltration within the TME. In addition, *LINK-A* induction has been associated with downregulation of β-2M and MHC-I expression in TNBC cells, via degradation of peptide loading complex (PLC), thus resulting in insufficient tumor antigen presentation by cancer cells and CTL unresponsiveness [84]. Moreover, three lncRNAs, myocardial infarction associated transcript (*MIAT*), *LINC01297* and MYLK antisense RNA (*MYLK-AS1)*, are included, together with four miRNAs (hsa-miR-200a-3p, hsa-miR-455-5p, hsa-miR-192-5p, has-miR-215-5P), in the underlying regulatory network of Ubiquitin Associated and SH3 Domain Containing B (UBASH3B), a protein which is involved in the infiltration of several immune cell types (macrophages, neutrophils, B and DCs, CD4+ and CD8+ T lymphocytes), in the TME of PC [85]. 

Furthermore, FOXC1-mediated overexpression of lncRNA *LINC00301* in NSCLC cells is associated with an increase in Treg and a decrease in CD8+ T cell populations, through activation of the FOXC1/*LINC00301*/EZH2/EAF2/pVHL/HIF1α and FOXC1/*LINC00301*/miR-1276/HIF1-α pathways [86]. Concomitantly, overexpression of lncRNA *RP11-323N-12.5* in both GC cells and associated TILs promotes Treg cell differentiation and immunosuppression by upregulating Yes1 associated transcriptional regulator (YAP1) [87]. In contrast, lncRNA FOXF1 adjacent non-coding developmental regulatory RNA (*FENDRR*), by acting as a sponge to miR-423-5p in HCC cells, was able to upregulate growth arrest and DNA damage inducible beta (GADD45B), which in turn could inhibit Treg-mediated HCC immune escape [88]. Likewise, expression of lncRNA cancer susceptibility candidate 2c (*CASC2c*) in glioblastoma (GBM) platform was associated with reduced M2 subtype macrophage polarization via suppression of the expression and secretion of the TAM-inducer, factor X (FX) [89]. 

Recent findings provide evidence for a novel activity of tumor-derived exosomal lncRNAs in interacting with immune cells, especially macrophages and Tregs, and regulating their immunological function. LncRNA small nucleolar RNA host gene 16 (*lnc-SNHG16)* detected in breast cancer-derived exosomes has been linked to CD73+ γδ1 Treg cell induction and consequently to TME immunosuppression. The underlying mechanism involves contact-free transmission of *lnc-SNHG16* from the indicated exosomes to γδ1 Τ cells where it upregulates CD73 expression in a miR-16-5p/TGF-β1/Smad5-dependant manner [90]. In addition, overexpression of lncRNA ribonuclease P RNA component H1 (*RPPH1*) in CRC promotes tumor cell proliferation and metastasis by interacting with the epithelial to mesenchymal transition (EMT)-inducer tubulin beta 3 class III (TUBB3) and facilitating exosome-mediated macrophage M2 polarization [91]. Similarly, HCC-derived exosomal lncRNA *TUC339* has been reported to regulate both macrophage activation and M1/M2 polarization [92]. The lncRNA X-Inactive Specific Transcript (*XIST*) is known for its regulatory role in breast cancer brain metastases (BCBM), yet its immunostimulating functions have only recently been discovered. Loss of *XIST* in mammary glands of mice not only promoted tumor cell proliferation, EMT and brain metastases, but also augmented secretion of exosomal miRNA-503, which triggered M1-M2 polarization of microglia with consequent induction of immune suppressive cytokines that inhibited T cell proliferation and function [93]. 

Expression of another BCBM regulator, the lncRNA associated with BCBM (*lnc-BM*), in BC cells has been associated with recruitment of macrophages in the brain via STAT3-dependent induction of C–C motif chemokine ligand 2 (CCL2), resulting in IL-6 accumulation and feedback hyperactivation of the *lnc-BM*/JAK2/STAT3 loop and hence further BCBM enhancement [94]. Likewise, lncRNA lymph node metastasis associated transcript 1 (*LNMAT1*), known as regulator of lymph node metastasis, activates in bladder cancer (BLCA) the transcription of CCL2, which facilitates recruitment of macrophages into the tumor mass and promotion of lymphatic metastasis via vascular endothelial growth factor (VEGF)-C secretion [95]. The overexpression of one of the most-studied lncRNAs, HOX transcript antisense RNA (*HOTAIR*), is a hallmark in many cancer types, contributing among others in tumor resistance, to immune-mediated cytotoxicity [96]. *HOTAIR*, the antisense strand transcript of the HOXC locus, mainly functions as a gene silencing regulator by cooperating with chromatin-modifying complexes [97]. On this motif, high levels of *HOTAIR* in HCC positively correlate with recruitment of macrophages and MDSCs to the TME, through CCL2 induction [98]. Finally, potent inducers of M2 polarization have been reported to be the lncRNAs LIN00662, RP11-361F15.2 and GMA-AS1 expressed in HCC, osteosarcoma, NSCLC and BC, respectively, via different molecular mechanisms [56,57,99,100].

### 3.2. Regulation of T Cell Function-Associated Immune Checkpoints by lncRNAs in Cancer

The unbalanced expression of lncRNAs in immune and tumor cells may also modify tumor immunosurveillance, via mechanisms that involve stimulation or suppression of various ‘breaks’ during the ‘cellular’ immune cell activation (priming phase) and response (effector phase). Inhibitory receptors, also known as immune checkpoints, and their ligands can be found on a wide range of T lymphocytes and cancer types, respectively [106]. Overexpression of inhibitory receptors, including programmed cell death protein-1 (PD-1), cytotoxic T-lymphocyte-associated protein 4 (CTLA-4) and T cell immunoglobulin mucin 3 (TIM-3), on T cells of cancer patients has been shown to counteract with co-stimulatory cell signals, leading to progressive loss of their activation potential and effector functions, exhaustion and eventually deletion [107,108,109]. LncRNAs affecting the expression of immune checkpoints are listed in Table 3 along with their underlying molecular mechanisms.

In this context, upregulation of the lncRNA nuclear-enriched autosomal transcript 1 (*NEAT1*) in PBMCs of HCC patients reduces CD8+ T cell survival and cytotoxicity, through modifications in the miR-155/Tim-3 axis. *NEAT1* causes augmentation of Tim-3 expression via interaction with miR-155, a negative regulator of Tim-3. Tim-3 upregulation in turn results in loss of CD8+ T cell cytolytic activity and their death, allowing HCC cell immune escape, invasion and metastasis [110]. Similarly, the overexpression of lncRNA *lnc-Tim3* in HCC-derived infiltrating CD8+ T lymphocytes appears to play a vital role in exacerbating their exhaustion, while preventing their apoptosis [111,112,113]. The underlying mechanism involves direct binding of *lnc-Tim3* to Tim-3 that prevents HLA-B-associated transcript 3 (Bat3) attachment and downstream activation of the Lck/NFAT1/AP-1 signaling. Lack of appropriate signaling leads to nuclear localization of Bat3 and enhancement of p-300-dependent p53 and RelA transcriptional activation of anti-apoptotic genes, including MDM2 and Bcl-2, therefore contributing to the survival of Tim-3+ exhausted CD8+ TILs [111].

Overexpression of lncRNA *MALAT1* in diffuse large B cell lymphoma (DLBCL) causes effector T cell apoptosis and anergy, by upregulating PD-L1 expression on cancer cells. The underlying molecular mechanism involves direct binding of *MALAT1* to miR-195, a suppressor of PD-L1 mRNA translation, thus allowing overexpression of PD-L1 on cancer cells. PD-L1 interacts with PD-1 and CD80 on CD8+ T cells, leading to the inhibition of their expansion, activation and cytotoxicity and consequently to tumor immune-escape [114]. Furthermore, *MALAT1* exerts similar effects on PD-L1 expression in NSCLC cells by modulating miR-200a-3p function [115]. Apart from improving malignant cell propagation, migration and EMT, another lncRNA, the small nucleolar RNA host gene 14 (*lnc-SNHG14*), also protects DLBCL cells from immune-mediated cytotoxicity. Briefly, overexpressed *lnc-SNHG14* in DLBCL acts as a miR-5590-3p sponge to facilitate de-repression of the zinc finger E-box binding homeobox 1 (ZEB1) translation. ZEB1 upregulation promotes the transcriptional activation of *lnc-SNHG14* and PD-L1, which in turn inhibits the activation of CD8+ T cells and induces their apoptosis by binding to the PD-1 molecules [116].

Likewise, overexpression of lncRNA urothelial cancer associated 1 (*UCA1*) in intestinal gastric cancer has been associated with reduction in effector T cell cytotoxicity and viability, via PD-L1 upregulation, mediated by *UCA1*-dependant ‘sponging’ of its translational repressors miR26a/b, miR-214 and miR-193a [117]. In pancreatic cancer models, high levels of lncRNA *LINC000473* reinforce tumor immunoescape by targeting another PD-L1 suppressor, miR-195-5p. PD-L1 upregulation in turn results in eliminated CD8+ T cell cytotoxicity and advanced apoptosis by induction of the pro-apoptotic protein Bcl-2-associated X protein (BAX) and inhibition of the anti-apoptotic factor Bcl-2. *LINC000473* further contributes to a decrease in the production of IFN-γ and IL-4 by CD8+ T cells, while it increases IL-10 secretion [118]. Contrarily, low expression of the lncRNA T cell leukemia/lymphoma 6 (*TCL6*) in breast cancer has been associated with worse prognosis in progesterone receptor (PR)-negative patients, attributed in part to tumor immune-escape, via regulation of immune-related pathways such JAK/STAT cascades. Specifically, *TCL6* was positively correlated with tumor infiltration with immune cells, including CD8+ and CD4+ T lymphocytes, neutrophils, B and DCs, as well as with the expression of PD-1, PD-L1, PD-L2 and CTLA-4 immune checkpoint molecules [119]. 

Moreover, lncRNA growth arrest-specific transcript 5 (*GAS5*) is a key player in maintaining an immunosuppressive microenvironment in CRC by regulating the expression of vascular endothelial growth factor A (VEGF-A) and IL-10 in cancer cells, via modulation of NF-κB and extracellular signal-regulated kinase 1/2 (ERK1/2) pathways, respectively [120]. These *GAS5*-mediated actions positively regulate the expression of inhibitory checkpoint molecules on CD8+ T cells and promote Treg differentiation, while they inhibit DCs maturation and antigen cross-presentation to T cells [121,122,123,124]. LncRNA small nucleolar RNA host gene 20 (*lnc-SNHG20*) induces p-ATM, p-JAK1/2 and PD-L1 in esophageal squamous cell carcinoma (ESCC), resulting in ESCC progression and metastasis [125], whereas upregulation of lncRNA NK2 homeobox 1 antisense RNA 1 (*NKX2-1-AS1*) in human lung adenocarcinoma (LUAD) negatively regulates CD274/PD-L1 axis and cancer cells migration [126]. Furthermore, lncRNA HOXA distal transcript antisense RNA (*HOTTIP*) enhances IL-6 expression to potentiate immune escape of ovarian cancer cells by upregulating PD-L1 in neutrophils [127], while expression of lncRNA actin filament associated protein 1 antisense RNA1 (*AFAP1-AS1*) in infiltrating lymphocytes in nasopharyngeal carcinoma (NPC) is positively correlated with expression of the immune escape receptor PD-1 on CTLs, distant metastasis and poor prognosis [128]. Finally, the expression of lncRNA microRNA 155 host gene (*MIR-155HG*) in a variety of tumor types, including skin cutaneous melanoma, cholangiocarcinoma, lung adenocarcinoma, glioblastoma multiform, kidney renal clear cell carcinoma, glioma and head and neck squamous cell carcinoma appears to significantly interfere with immune cell infiltration within TME and expression of immune breaks, such as PD-1, PD-L1, CTLA-4, LAG3 and TIM-3, hence affecting tumor responsiveness to immune checkpoint inhibitors [129].

### 3.3. Regulation of Anti-Tumor NK-Mediated Cytotoxicity by lncRNAs 

The immune escape of cancer cells may also be promoted by alterations in the expression patterns of lncRNAs associated with NK cell activation and cytotoxic activity as well as with their potency to infiltrate the TME. Some of these lncRNAs are solely expressed by NK cells (listed in Table 1), while others by tumors (listed in Table 2). 

Among several cancer types, *HOTAIR* overexpression in leukemia has been associated with tumor immune-escape through increased activation of the Wnt/β-catenin signaling pathway. Hyperactivation of this cascade in leukemic patients is considered a potent suppressor of both innate and adaptive immunity, as it is positively correlated with a reduction in NK cell activity, a decreased CD4+/CD8+ T subset ratio and a decline in cytokine release in peripheral blood, as well as in Ig production by B-lymphocytes [101]. In addition, in early-stage HCC, the expression patterns of a network of five lncRNAs, namely *AATK-AS1*, *C10orf91*, *LINC000162*, *LINC00200* and *LINC00501*, that are known to act as competing endogenous RNAs (ceRNAs), positively correlate with good tumor prognosis and overall patients’ survival, along with tumor infiltration by activated CD4+ memory T cells, NK and mast cells [102]. CeRNAs have been considered as the drivers in many diseases, including cancer, by interacting with miRNA response elements (MREs) found in both coding and non-coding RNAs, thus reducing the amount of miRNAs available to target mRNAs and relieving the miRNA repression on competing RNAs [16]. 

In contrast to IL-2-activated NK cells, which show high expression of lncRNA *GAS5* and intense IFN-γ release, lncRNA *GAS5* is downregulated in liver cancer patient-derived NK cells, contributing to reduced NK-mediated cytotoxicity and tumor immune-escape. The underlying mechanism involves direct interaction of lncRNA *GAS5* with miR-544, a translational repressor of runt-related transcription factor 3 (RUNX3). Induction of RUNX3 promotes the transcription of the natural cytotoxicity receptor 1 (NCR1) gene and the expression of the encoded protein NKp46, an activating receptor that stimulates INF-γ secretion. Hence, downregulation of lncRNA *GAS5* in the NK cells of liver cancer patients results in decreased natural cytotoxicity receptor NKp46 levels, a decline in INF-γ release and, eventually, in a reduction in NK cell cytotoxicity and CD107+ levels, thus facilitating tumor immune escape [79]. In this context, reduced expression of lncRNA *RP11-222K-16.2* in NK cells of acute myeloid leukemia (AML) patients eliminates NK cell differentiation via cis-acting regulation of a nearby gene that encodes for the eomesodermin protein, a transcriptional activator of genes related to NK development [80]. Similarly, IFNβ-induced exosomal lncRNA EPH receptor A6-1 (*linc EPHA6-1*) enhances NK cytotoxicity, by acting as a competing endogenous RNA (ceRNA) for hsa-miR-4485-5p, leading to overexpression of the natural cytotoxicity receptor NKp46 [103], while lncRNA IFNγ antisense 1 (*IFNG-AS1*) is associated with increased IFN-γ secretion from NK cells, that enhances their activity [81]. Finally, *lnc-CD56* positively regulates the expression of the NK surface marker CD56, thus promoting the polarization of CD34+ hematopoietic progenitor cells towards a NK cell phenotype [82]. 

Several lncRNAs may also interfere indirectly with NK anti-tumor activity and tumor immune escape by regulating the expression and shedding of MHC class I chain related A (MICA). MICA is one of the natural killer group 2, member D (NKG2D) ligands (NKG2DLs), produced by virus-infected or malignant cells and integrated into their cellular membrane. MICA can be recognized by NKG2D, an activating receptor located on the membrane of NK and other immune cells, while the MICA-NKG2D interaction can positively regulate host immune-surveillance against MICA-expressing cells [104]. Apart from the vast range of MICA polymorphisms, dysregulation of MICA expression in tumor cells might also help the later to evade immune-mediated cytotoxicity. For example, some tumor cells develop the capacity to discard MICA molecules off the cell surface using proteolysis, a process known as “MICA shedding”. Various miRNAs and lncRNAs seem to be associated with the regulatory mechanisms of MICA expression, such as the lncRNA *XXbac-BPG181B23.7* [104]. It is thus possible that changes in the expression patterns of lncRNAs like *XXbac-BPG181B237* could contribute to the immune toleration of tumor cells by altering MICA presentation (Table 2). 

In the same context, the circular lncRNA *circ_0000997* was found to be a central regulator of hypoxia-induced resistance of pancreatic cancer (PACA) cells to NK-mediated killing, via promoting MICA shedding from tumor cell membranes. Briefly, the hypoxic microenvironment in PACA can induce *circ_0000997* upregulation in malignant cells, which in turn sponges miR-153 and de-represses HIF-1α and a disintegrin and metalloproteinase domain 10 (ADAM10) translation. The elevated expression of HIF-1α and ADAM10 promote the shedding of MICA from the membrane of PACA cells (mMICA), thus increasing the levels of the soluble MICA (sMICA) molecules, which further bind to NKG2D receptors on NK cells. This interaction in turn, enhances the internalization and degradation of the receptor, hence triggering reduction in NK cell activity [105] (Table 2).

## 4. LncRNAs and Tumor Response to Immunotherapy

The dysregulated expression of a wide range of lncRNAs has been reported to affect not only the endogenous immune surveillance against cancer, but also the tumor response to exogenous immunotherapy. Although the relevant studies are still limited and mainly focused on Ab-mediated immunotherapy, it cannot be ruled out that lncRNAs might also be involved in the regulation of immune responses to adoptive cell transfer (ACT). A recent cohort study of 348 patients with bladder cancer and 71 patients with melanoma, correlated lncRNA-based immune subtypes that are associated with overall survival (OS) and patient response to cancer immunotherapy, aiming to produce a lncRNA score for multi-omic panels in precision immunotherapy. The findings revealed four distinct classes of lncRNA-based immune subtypes with significant differences in OS and response to immunotherapy, while the one with the greatest OS included the immune-active class which was characterized by the immune-functional lncRNA signature associated with high CTL infiltration [130]. Table 4 contains a list of lncRNAs reported to regulate tumor response to conventional Ab-mediated immunotherapy.

Among the mAbs currently used for Ab-based cancer immunotherapy, trastuzumab, a human epidermal growth factor receptor (HER2) inhibitor used for early-stage and metastatic HER2+ BC and GC treatment [131], compiles a long list of lncRNAs whose expression dysregulation seems to interfere with its action. Overexpression of lncRNA activated by TGF-β (*lnc-ATB*) in the SKBR-3 cell line and corresponding BC tissues has been associated with their resistance to trastuzumab, through a sponging effect on miR-200c that results in the upregulation of EMT-inducers ZEB1 and zinc finger protein 217 (ZNF-217) and the dysregulation of the TGF-β signaling [132]. In contrast, reduced levels of the lncRNA *GAS5* in the same cell and tissue models enhanced tumor cell proliferation and resistance to trastuzumab via modulation of the miR-21/PTEN/mTOR axis [133]. LncRNA-small nucleolar RNA host gene 14 (*lnc-SNHG14*) is another example of lncRNA whose elevated levels in HER2+ BC cells and their secreted exosomes has been associated with diminished tumor response to trastuzumab. Although the exact underlying molecular mechanism of resistance is still unknown, the involvement of the apoptosis-related molecules Bcl-2/Bax is considered possible [134].

Primary or acquired resistance has been also reported for cetuximab, a therapeutic mAb that binds to EGF receptor and induces its degradation in CRC patients with metastatic disease. The presence of abnormally increased levels of lncRNA *UCA1* in CRC cells and their secreted exosomes has been proposed as potential clinical biomarker for dictating tumor resistance to cetuximab [135]. In TNBC patients, the lack of hormonal receptors excludes the treatment option with anti-hormone inhibitors. Although the Ab-based immunotherapy with pembrolizumab, is currently employed as an alternative treatment option, TNBC patients showing resistance to anti-PD-1 treatment, are also unresponsive to pembrolizumab [136]. In these patients the overexpression of lncRNA *LINK-A* has been strongly correlated with increased resistance to PD-1 blockade by immune checkpoint inhibitors [84]. Similarly in patients with NSCLC, among other cancers, receiving anti-PD-1 immunotherapy, lncRNA *RP11-705C-15.3* levels have been considered a putative prognostic factor for the treatment outcome [137]. Durvalumab, an antibody used for the treatment of NSCLC and BLCA, acts by blocking the interaction of PD-L1 with PD-1 and CD80. Patients treated with Durvalumab showed a higher survival rate, when they had augmented expression level of lncRNA *RP11-291B-21.2*, thus suggesting that *RP11-291B-21.2* can be used as a potential biomarker of PD-1/PD-L1 blockage [138].

## 5. Cancer Stem Cells, lncRNAs and Tumor Immune Escape

The ability of cancer stem cells (CSCs) to evade immune destruction was initially proposed by the hierarchical model of cancer along with the cancer immune-editing theory [139]. The small subpopulation of CSCs consists of pluripotent and long-living cancer cells that are held responsible for tumor initiation, metastasis, recurrence and drug resistance [140,141,142]. Along with their ability to initiate neoplastic growth, regardless of cancer immunosurveillance, CSCs persist in latent tumors by forming specialized niche-constrained cell reservoirs which, in contrast to niche-independent immunogenic daughter cells, are immune-privileged [143]. 

CSCs evade immune-mediated destruction and actively suppress innate and adaptive anti-tumor immune responses by expressing, diverse from non CSCs, intracellular, membrane-bound and soluble factors that allow them to survive in their niche and interact with a broad range of immune cells [144,145,146]. For example, CSCs in different tumor types show dysregulated expression of several anti-apoptotic and survival-inducing proteins such as Bcl-2, Bcl-xL and surviving. They also characterized by a distinct repertoire of secreted cytokines and other molecules, including IL-4, IL-6, IL-10, PGE2 and FasL that protect them from immune-mediated apoptosis triggered by effector T and NK cells, or by immunotherapeutic antibodies [143,146,147,148,149,150,151,152,153]. In addition, the CSC-immune cell crosstalk along with CSC-secreted chemotactic factors and immunosuppressive cytokines, such as TGF-β, [143,154] advance the establishment of an immunosuppressive TME either by fostering tumor infiltration by a wide range of immunosuppressive immune cell subpopulations, including MDSCs, Tregs and TAMs, or/and by inhibiting the recruitment and activities of effector immune cells such as NK cells, CTLs and T cells (reviewed by Bayic and Lathia, 2021 [145] and Vahidian et al., 2019) [146]).

Studies on the multifaceted interactions between CSCs and the immune system have revealed that CSCs prevent the differentiation of naïve CD8+ T cells into CTLs during the CD8+ T cell priming by APCs, via downregulating the expression of CD80 (B7.1) and CD86 (B7.2) ligands responsible for secondary signals, while elevating levels of the inhibitory co-stimulatory ligand PD-L1 [155]. The priming of naïve CD8+ T cells by APCs may be further inhibited by CSC-mediated induction of CTLA-4 on T cells, a vital negative regulator of T cell response, leading to T cell exhaustion and dysfunction [155,156,157]. Accordingly, CSCs are able to suppress their recognition by activated CTLs through elimination of the surface expression of MHC I molecules [158] and inadequate presentation of TAAs or tumor specific antigens (TSAs), which are also less immunogenic than those of non CSCs [143,146,147,148]. Finally, CSCs from different cancer types show abnormally elevated levels of inhibitory NK cell receptor ligands that protect them against destruction by innate anti-tumor immunity [143,144,146,147].

Although the direct impact of lncRNAs repertoire in the immunomodulatory potential of CSCs has not been directly exploited so far, it is, however, clear that the TME affects the lncRNA-regulated therapy resistance in CSCs [159]. Hypoxia, a major feature of TME, has been shown to promote cancer stemness and associated resistance to apoptosis by modulating the non-coding transcriptome, including the lncRNAs runt-related transcription factor 1 Intronic Transcript 1 (*RUNX1-IT1*) and hypoxia-associated lncRNA (*HAL*) in HCC and BC models, respectively [159,160,161,162]. In addition, the crosstalk of tumor cells with immunosuppressive immune cell components of TME, like TAMs, has been demonstrated in HCC to mediate cancer stemness and resistance to apoptotic signals through modulation of the lncRNA *H19*/miR-193b/MAPK1 axis [163]. 

Nevertheless, signaling pathways known as “hallmarks” for CSC functions, have now been identified as critical regulators of the tumor immune response. Among them the activation of Wnt/β-catenin signaling has been positively associated with cancer stemness, drug resistance and immune exclusion across several non-T cell-inflamed human tumors, thus suggesting a negative impact of this pathway in immune cell infiltration into TMEs [164,165,166,167,168]. Studies in diverse cancer types have revealed that CSC-associated lncRNAs are directly or indirectly involved in tumor resistance to CSC elimination by apoptosis, via regulating the Wnt/β-catenin cascade [159]. In this context, in GBM CSCs, down regulation of *lincRNAp21*, a negative regulator of β-catenin, suppresses CSC apoptosis [169], whereas in NSCLC cells the inhibition of lncRNA *NEAT1*, an lncRNA responsible for CSC enrichment, restrained the stemness traits and re-sensitized cells to apoptosis, via de-activation of the Wnt pathway [170]. Mechanistically, LncRNAs mediate the regulation of Wnt/β-catenin signaling, either at transcriptional level or by competing the action of pathway inhibitors [159].

Similarly, Notch signaling, elicits a CSC phenotype that contributes among others to CSC dormancy and resistance to immune destruction, via supporting an immunosuppressive microenvironment [171,172]. The Jagged-1-Notch4 axis is highly expressed in endocrine resistant breast CSCs and this activity has been correlated with the differentiation and polarization of macrophages to TAMs and eventually to decreased anti-tumor immune responses [173]. Notch-induced lncRNAs, such as *LUNAR1*, associates with reduced apoptosis of CRC cells and overall patient survival [174], while Notch-inducing lncRNAs, like prostate cancer-upregulated long noncoding RNA 1 (*PlncRNA-1*) function as an apoptosis inducer in glioma tumors [175]. Similarly, lncRNA antisense non-coding RNA in the INK4 locus (*ANRIL*) inhibits apoptosis and enhances progression of GC cells by targeting among others the Notch pathway [176,177].

In conclusion, although the literature lacks reports that demonstrate a direct link of lncRNAs involved in the regulation of CSC features, with CSC immune response or evasion, it is highly suggestive that signaling pathways important for both CSC functions and immune destruction may be downstream targets or upstream regulators of critical lncRNAs.

## 6. Future Perspectives

Early cancer diagnosis is critical for treatment success. Until recently, the era of cancer biomarkers was mainly enriched only by coding gene products. However, over the past few years, the focus on biomarker development has been shifted to previously thought “genetic debris”, now known as non-coding RNAs, that have broadened the diagnostic and prognostic panels of a vast variety of human cancers. LncRNAs are key players in the regulation of cellular events and the pathophysiology of several diseases, including cancer and its associated characteristics [21]. LncRNAs are considered great candidates for diagnostic and prognostic biomarkers, given that their levels can be indicative of the stage of the disease, while they are easily detectable in body fluids, such as saliva, blood, plasma and urine [178,179,180]. LncRNAs can be also detected in exosomes or inside apoptotic bodies conjugated with RNA-binding proteins, thus avoiding RNase degradation [181,182]. In addition, their property to exhibit tissue-specific expression patterns makes them ideal targets for therapy [21]. Furthermore, the high sensitivity and specificity of lncRNAs undoubtedly make them important research tools for cancer diagnosis, prognosis and therapeutic targeting [183].

Although the translational potential of lncRNA targeting in clinic is still in its infancy, there are several quite novel strategies that can potentially be proved effective for specific lncRNA manipulation. These strategies mainly include post-transcriptional degradation of lncRNAs, modification of lncRNA genes and loss of lncRNAs’ function [184]. The post-transcriptional degradation may be facilitated either by double-stranded RNA-mediated interference (RNAi) or single-stranded antisense oligonucleotides (ASOs) [184]. Although ASOs have already been used in clinical trials as a promising therapeutic strategy with good results, RNAi faces excessive difficulties in preclinical studies, due to ineffective delivery and low bioavailability [184,185]. Alternatively, modifications of lncRNA genes can be induced by application of the novel clustered regularly interspaced short palindromic repeats (CRISPR)-Cas system that is able to silence lncRNA expressing loci and block lncRNA transcription by merging Cas9 with transcriptional repressors that target the specific gene promoter [186,187]. Moreover, blocking of lncRNA-protein interactions or secondary/tertiary lncRNA formation structures can be achieved by small molecule inhibitors, or specific ASOs that can recognize and bind to lncRNAs [184].

Other major challenges that need to be overcome for applying therapeutic lncRNA targeting approaches in clinical practice, especially in the field of oncology and beyond, is the identification and characterization of the right lncRNA candidates, as well as the deep understanding of their role in cellular functions associated not only with initiation and progression of cancer, but also with individual characteristics of the tumor status, such as the acquisition of resistance to immunosurveillance, immunotherapy or chemotherapy [15]. It is also critical to select and characterize lncRNAs with crucial involvement in specific cancer type, subtype, or subpopulation like CSCs; however there is still a limited number of lncRNA-associated structural and functional data between normal and cancerous tissues [188].

Summarizing, over the last decade substantial effort has been made towards the identification of several lncRNAs that can potentially be implemented as diagnostic and prognostic biomarkers for both carcinogenesis and tumor resistance to endogenous and exogenous immune-mediated cytotoxicity. Nevertheless, the clinical application of lncRNA-based therapeutics is still in its infancy. Key challenges, including lncRNA specificity, delivery and tolerability issues, should be areas of extensive research. This, together with new technological advances, could enable the effective translation of newly developed lncRNA therapeutics.

## Figures and Tables

**Table 1:** Regulation of anti-tumor immuno-mediated cytotoxicity by lncRNAs expressed in cells of adaptive and innate immunity.

LncRNA	Expressing Cell Population	Cancer Type ^#^	Action	Effect	Molecular Targets	Ref.
*SNHG1*	CD4+ TILs	BC	IS	Induces Treg polarization Suppresses CTL activity Induces CTL apoptosis	miR-448 /IDO	[38]
*EGFR*	CD4+ TILs	HCC	IS	Induces Treg polarization Suppresses CTL propagation and activity	EGFR/AP1/ NFAT1/ Foxp3	[39]
*INSR*	BM-infiltrating CD4+ T cells	Pediatric T-ALL	IS	Increases Treg numbers Suppresses CTL propagation and activity	INSR/PI3K/ AKT	[40]
*linc-* *POU3F3*	Tregs	GC	IS	Increases Treg distribution	TGF-β/ SMAD2/3	[41]
*Flart*	Tregs	-	IS	Treg induction	FoxP3	[42]
*Flicr*	Tregs	-	IM	Impairs Treg activity	FoxP3	[43]
*Linc-* *MAF-4*	CD4+ T cells	-	IS	Promotes T cell differentiation towards a Th2 phenotype	MAF	[44]
*SGK1*	TILs	GC	IS	Promotes Th2 and Th17 differentiation Inhibits Th1 differentiation	SGK1/ Jun-B	[45]
*NKILA*	CD8+ TILS Th1 TILS	LUAD, BC	IS	Promotes CD8+ T cell AICD	STAT1/NF-κB	[46]
*Morrbid*	CD8+	-	IM	Induces CD8+ T cell expansion and survival	Bcl2111/PI3K/AKT	[47]
*GM16343*	CD8+ T cells	CRC	IM	Increases CD8+ T cell cytotoxicity	IL-36	[48]
*DC*	DCs	-	IM	Promotes DC differentiation and T cell stimulation	STAT3	[51,52]
*Dpf3*	DCs	-	IS	Restrains CCL7-mediated DC migration to dLNs	HIF-1α	[53]
*GNAS-AS1*	Mφ	NSCLC BC	IS	Induces M2 polarization	MIR4319/NECAB3 and *GNASAS1*/ miR-433-3p/GATA3	[56,57]
*LIFR-AS1*	Μφ	OS	IS	Promotes CC proliferation, and invasion Restrains CC apoptosis	miR-29a/ NFIA	[62]
*Sbf2-AS1*	Μφ	PACA	IS	Cancer progression	miR-122-5p/XIAP	[63]
*NIFK-AS1*	Μφ	UCEC	IM	Inhibits M2 Polarization	miR-146a	[64]
*COX-2*	Μφ	HCC	IM	Inhibits M2 Polarization	Not stated	[65,66]
*CCAT1*	Μφ	PRAD	IM	Inhibits M2 Polarization	miR148a/ PKCζ	[67]
*XIST*	Μφ	LUAD	IS	Induces M2 polarization	TCF-4	[58]
*P21*	Μφ	BC	IS	Inhibits M1 polarization	MDM2/p53/NFκB/ STAT3	[59]
*ANCR*	Μφ	GC	IS	Inhibits M1 polarization	FoxO1	[60]
*MM2P*	BMDM	OS ^@^ OV	IS	Induces M2 polarization	STAT6	[61]
*CHOP*	MDSCs	MM, LUAD, BC	IS	Represses CD4+ and CD8+ T cell proliferation	CHOP/C/EBPB	[74]
*Olfr29-ps1*	Mo-MDSCs	MM, CRC, READ	IS	Promotes MDSC differentiation and T cell inhibition	miR-214-3p/MyD88/M6A	[75]
*RNCR3*	MDSCs	MM	IS	Promotes MDSC expansion, differentiation and immunosuppressive function	miR-185-5p/Chop	[76]
*Pvt1*	G-MDSCs	LUAD	IS	Promotes MDSC-mediated immunosuppression Inhibits T cell responses	Arg1 and ROS	[77]
*RUNXOR*	MDSCs	LUAD	IS	Promotes MDSC-mediated immunosuppression	RUNX1/ Arg1	[78]
*HOTAIRM1*	MDSCs	LUAD	IM	Inhibits MDSC-mediated immunosuppression	HOXA1	[69]
*MALAT1*	MDSCs	LUAD	IM	Inhibits MDSC expansion and accelerates CTL proportion	Arg1	[73]
*GAS5*	NK	HCC	IM	Increases NK cytotoxicity	miR-544/RUNX3	[79]
*RP11-222K-16.2*	NK	AML	IM	Increases NK cell differentiation and CD8+ T cell cytotoxicity	Eomeso- dermin	[80]
*IFNG-AS1*	NK	-	IM	Increases NK cell activity	IFN-γ	[81]
*Lnc-CD56*	NK	-	IM	CD34+ hematopoietic progenitor cell polarization to NK cell phenotype	CD56	[82]

Abbreviations used: IS, Immunosuppression; IM, Immunomodulation; AICD, Activation induced cell death; BM, Bone marrow; CC, Cancer cell; Linc, Long intragenic non coding; Mφ; DCs, Dendritic cells; dLNs, Distant lymph nodes; Μφ, Macrophages; NK, Natural Killer cells; MDSCs, Myeloid-derived suppressor cells; mo-MDSCs, Monocytic-myeloid-derived suppressor cells; G-MDCs, Granulocytic-myeloid-derived suppressor cells; TILs, Tumor infiltrating lymphocytes; Tregs, Regulatory T cells; CTLs, cytotoxic T lymphocytes; BMDM, Bone marrow derived macrophages. ^#^ NCI-based TCGA-based abbreviations available online: https://gdc.cancer.gov/recources-tcga-users/tcga-code-tables/tcga-study-abbreviations (accessed on 13 October 2021): UCEC, Uterine corpus endometrial carcinoma; MM, Malignant melanoma; OS, Osteosarcoma; HCC, Hepatocellular carcinoma; BC, Breast Cancer; CRC, Colorectal Cancer; AML, Acute myeloid leukemia; OV, Ovarian serous cystadenocarcinoma; NSCLC, Non-small cell lung cancer; PRAD, Prostate adenocarcinoma; T-ALL, T cell acute lymphoblastic leukemia; GC, Gastric cancer; READ, Rectum adenocarcinoma; LUAD, Lung adenocarcinoma; PACA, Pancreatic cancer. ^@^ Murine osteosarcoma.

**Table 2:** Regulation of anti-tumor immuno-mediated cytotoxicity by lncRNAs expressed by cancer cells.

LncRNA	Cancer Type ^#^	Action	Effect	Molecular Targets	Ref.
*lnc-sox5*	CRC	IS	Induces Treg differentiation Suppresses CTL activity and infiltration into TME	IDO1	[83]
*LINK-A*	ΤΝΒC	IS	Suppresses MHCI expression in TMBCs Suppresses APC and CTL infiltration into TME	PIP3/GPCR/PKA	[84]
*MIAT*, *LINC01297*, *MYLK-AS1*	PRAD	IS	Inhibits TME infiltration by IM immune cells	UBASH3B	[85]
*LINC00301*	NSCLC		Induces Tregs Decreases CD8+ T cells	FOXC1/*LINC00301*/EZH2/EAF2/pVHL/HIF1α and FOXC1/*LINC00301*/ miR-1276/HIF1α	[86]
*RP11-323N-12.5*	GC	IS	Enhances Treg differentiation	YAP/TAZ/TEAD	[87]
*FENDRR*	HCC	IM	Inhibits Treg activation and proliferation	miR-423-5p/GADD45B	[88]
*CASC2c*	GBM	IM	Reduces M2 polarization	FX	[89]
*SNHG16*	BC	IS	Induces CD73+Vδ1 Tregs	miR-16-5p/ TGF-β1/Smad5	[90]
*RPPH1*	CRC	IS	Induces M2 polarization	TUBB3	[91]
*TUC339*	HCC	IS	Regulates macrophage activation and M2 polarization	N/A	[92]
*XIST*	BCBM	IS	Induces M2-polarization of microglia Inhibits T cell proliferation	c-Met/MSN, miRNA-503	[93]
*lnc-BM*	BCBM	IS	Regulates Μφ recruitment to the brain	JAK2/STAT3/ CCL2	[94]
*LNMAT1*	BLCA	IS	Induces Mφ recruitment and lymphatic metastasis	CCL2, VEGF-C	[95]
*HOTAIR*	HCC	IS	Induces Μφ and MDSCs proliferation and recruitment to TME	CCL2	[98]
*LIN00662*	HCC	IS	Induces M2 polarization	WNT3A/ Wnt/β-catenin	[99]
*RP11-* *361F15.2*	OS	IS	Induces M2 polarization	miR-30c-5p/ CPEB4	[100]
*GNA-AS1*	NSCLC, BC	IS	Induces M2 polarization	MIR4319/NECB3 and *GNASAS1*/miR433-3p/GATA3	[56,57]
*HOTAIR*	Τ-ALL	IS	Decreases CD4+/CD8+ T cell ratio, NK cell activity cytokine release, Ig production by B cells	Wnt/ β-catenin	[101]
*AATK-AS1*, *C10orf91*, *LINC000162*, *LINC00200 LINC00501*	Early stage HCC	IM	Increases the proportion of activated CD4+ memory T cells, NK and mast cells	Acts as ceRNA	[102]
*Linc* *EPHA6-1*	NSCLC	IM	Increases NK cell cytotoxicity	miR-4485-5p/ NKp46	[103]
*XXbac-BPG181B23.7*	HCC	IS	Reduces NK cell activity	MICA	[104]
*circ_* *0000997*	PACA	IS	Reduces NK cell activity	miR153/ HIF1A/ ADAM10/ MICA/ NKG2D	[105]

Abbreviations used: IS, Immunosuppression; IM, Immunomodulation; N/A, not assessed; MDSCs, Myeloid-derived suppressor cells, NK, Natural killer cells; ceRNA, competing endogenous RNA; Μφ, Macrophages; CTL, Cytotoxic T lymphocytes; TILs, Tumor infiltrating lymphocytes; TME, Tumor microenvironment; Tregs, Regulatory T cells. ^#^ NCI-based TCGA-based abbreviations available online: https://gdc.cancer.gov/recources-tcga-users/tcga-code-tables/tcga-study-abbreviations (accessed on 13 October 2021): OS, Osteosarcoma; HCC, Hepatocellular carcinoma; BC, Breast Cancer; CRC, Colorectal Cancer; NSCLC, Non-small cell lung cancer; PRAD, Prostate adenocarcinoma; T-ALL, T cell acute lymphoblastic leukemia; GC, Gastric cancer; PACA, Pancreatic cancer; BCBM, Breast cancer brain metastasis; GBM, Glioblastoma multiform; BLCA, Bladder cancer; Triple-negative breast cancer (TNBC).

**Table 3:** Regulation of T cell function-associated immune checkpoints by lncRNAs.

LncRNA	Expressing Cell population	Cancer Type ^#^	Action	Effect	Molecular Targets	Ref.
*NEAT1*	PBMCs	HCC	IS	Induces Tim-3 Suppresses CTL function and survival	miR-155/ Tim-3	[110]
*lnc-Tim-3*	CD8+ TILS	HCC	IS	CD8+ T cell exhaustion	Bat3/Lck/ NFAT1/AP-1	[111]
*MALAT1*	CCs	DLBCL NSCLC	IS	Induces PD-L1 overexpression and CTL anergy	miR-195 miR-200a-3p	[114,115]
*SNHG14*	CCs	DLBCL	IS	Induces PD-L1 overexpression and CTL cell apoptosis	miR-5590-3p/ ZEB1	[116]
*UCA1*	CCs	GC	IS	Induces PD-L1 overexpression and eliminates CTL function and survival	miR26a/b, miR-214, miR-193a	[117]
*LINC* *000473*	CCs	PACA	IS	Induces PD-L1 overexpression and CTL anergy and apoptosis	miR-195-5p/ Bax/Bcl-2	[118]
*TCL6*	CCs	BC	IM	Induces TILs, PD-1, PD-L1, PD-L2 and CTLA-4 expression	JAK/STAT	[119]
*GAS5*	CCs	CRC	IS	Induces Tregs and immune checkpoints on T cells Inhibits DC maturation and antigen presentation	NF-κB Erk1/2	[120,121,122,123,124]
*SNHG20*	CCs	ESCC	IS	Induces PD-L1	p-ATM/ p-JAK	[125]
*NKX2-1-* *AS1*	CCs	LUAD	IM	Suppresses PD-L1	CD274	[126]
*HOTTIP*	Neutrophils	OV	IS	Induces PD-L1	STAT3	[127]
*AFAP1-* *AS1*	TILs	NPC	IS	Induces PD-1	N/A	[128]
*MIR-* *155HG*	CCs	SKCM CHOL LUAD GBM KIRC LGG HNSC HCC UVM		Induces PD-1, PD-L1, CTLA-4 Induces immune cell infiltration	N/A	[129]

Abbreviations used: IS, Immunosuppression; IM, Immunomodulation; N/A, not assessed; CTLs, Cytotoxic T lymphocytes; TILs, Tumor infiltrating lymphocytes; Tregs, Regulatory T cells; CCs, Cancer cells, DCs, Dendritic cells; PBMCs, Peripheral blood mononuclear cells; PD-1, Programmed cell death protein-1; PD-L1/2; Programmed death-ligand 1; CTLA-4, Cytotoxic T-lymphocyte-associated protein 4. ^#^ NCI-based TCGA-based abbreviations available online: https://gdc.cancer.gov/recources-tcga-users/tcga-code-tables/tcga-study-abbreviations (accessed on 13 October 2021): HCC, Hepatocellular carcinoma; BC, Breast Cancer; CRC, Colorectal Cancer; OV, Ovarian serous cystadenocarcinoma; NSCLC, Non-small cell lung cancer; GC, Gastric cancer; LUAD, Lung adenocarcinoma; PACA, Pancreatic cancer; NPC; nasopharyngeal carcinoma; ESCC, Esophageal squamous-cell carcinoma; DLBCL, Diffuse large B-cell lymphoma; SKCM, Skin cutaneous melanoma; CHOL, Cholangiocarcinoma; GBM, glioblastoma multiform; KIRC, Kidney renal clear cell carcinoma; LGG, Brain low grade glioma; HNSC, head and neck squamous cell carcinoma; UVM, Uveal melanoma.

**Table 4:** LncRNAs involved in tumor response to mAb-mediated immunotherapy.

LncRNA	Type of Cancer ^#^	Drug	Ab Target	Effect	Resistance Mediators	Ref.
*lnc-ATB*	BC	Trastuzumab	EGFR	Induces resistance	ZEB1, ZNF-217, miR-200c	[132]
*GAS5*	BC	Trastuzumab	EGFR	Augmented response	miR21/ PTEN/ mTOR	[133]
*SNHG14*	HER2+ BC	Trastuzumab	EGFR	Induces resistance	Bcl-2/Bax	[134]
*UCA1*	CRC	Cetuximab	EGFR	Induces resistance	N/A	[135]
*LINK-A*	TNBC	Pembrolizumab	PD-1	Induces resistance	N/A	[84,136]
*RP11-705C-15.3*	NSCLC	PD-1 checkpoint inhibitors	PD-1	Induces resistance	N/A	[137]
*RP11-291B-21.2*	NSCLC BLCA	Durvalumab	PD-L1	Augmented response	N/A	[138]

Abbreviations used: IS, Immunosuppression; IM, Immunomodulation; N/A, not assessed; CTLs, Cytotoxic T lymphocytes; TILs, Tumor infiltrating lymphocytes; Tregs, Regulatory T cells; CCs, Cancer cells, DCs, Dendritic cells; PBMCs, Peripheral blood mononuclear cells; PD-1, Programmed cell death protein-1. ^#^ NCI-based TCGA-based abbreviations available online: https://gdc.cancer.gov/recources-tcga-users/tcga-code-tables/tcga-study-abbreviations (accessed on 13 October 2021): BC, Breast Cancer; CRC, Colorectal Cancer; NSCLC, Non-small cell lung cancer; TNBC, Triple negative breast cancer; BLCA, Bladder cancer.
